# Construction and validation of an instrument for the assessment of care provided to people with suicidal behavior

**DOI:** 10.11606/S1518-8787.2019053000888

**Published:** 2019-05-02

**Authors:** Laura Maria Souza de Linhares, Patrícia Moita Garcia Kawakame, Daniel Henrique Tsuha, Albert Schiaveto de Souza, Ana Rita Barbieri

**Affiliations:** I Universidade Federal de Mato Grosso do Sul . Programa de Pós-Graduação em Saúde da Família . Campo Grande , MS , Brasil; II Universidade Federal de Mato Grosso do Sul . Curso de Graduação em Enfermagem. Instituto Integrado em Saúde . Campo Grande , MS , Brasil; III Secretaria de Estado de Saúde de Mato Grosso do Sul . Central Estadual de Regulação Assistencial . Campo Grande , MS , Brasil; IV Universidade Federal de Mato Grosso do Sul . Instituto de Biociências . Campo Grande , MS , Brasil; V Universidade Federal de Mato Grosso do Sul . Pró-Reitoria de Assuntos Estudantis. Campo Grande , MS , Brasil

**Keywords:** Suicide, Attempted, prevention & control, Surveys and Questionnaires, utilization, Patient Care Team, Health Knowledge, Attitudes, Practice, Validation Studies, Tentativa de Suicídio, prevenção & controle, Inquéritos e Questionários, utilização, Equipe de Assistência ao Paciente, Conhecimentos, Atitudes e Prática em Saúde, Estudos de Validação

## Abstract

**OBJECTIVE:**

To develop and validate an instrument for evaluating primary health care professionals’ assistance to people with suicidal behavior.

**METHODS:**

This was a methodological study, which began with a literature review, followed by the elaboration of an instrument. In its first version, the instrument had 34 items, divided into four domains: “professional characterization,” “professional perception ” “professional knowledge/abilities,” and “organization of the care network.” Contents were validated using the Delphi method. Semantic analysis was performed by college-educated primary health care professionals in greater and lesser strata of ability. For internal consistency analysis, Cronbach’s alpha coefficient was calculated. The study was conducted between January and December 2017.

**RESULTS:**

After four Delphi rounds, the instrument was successfully validated. In its final form, it is comprised of 50 items, divided into five domains: “professional characterization,” “professional sensibility,” “professional experience,” “professional knowledge/abilities,” and “organization of the care network.” Questions belonging to the last four domains have answers on a five-point Likert scale. In the semantic analysis, 93.6% of the evaluations were “good” and “very good.” The instrument’s general Cronbach alpha was 0.90.

**CONCLUSIONS:**

The final version of the instrument was able to fulfill its objectives. It is useful as a support for epidemiological research and planning of health actions. The evaluation of professional approaches to suicidal behavior is crucial for the organization of suicide assistance services in primary health care, and for the integration of services provided by different care units.

## RESUMO

**OBJETIVO:**

Desenvolver e validar um instrumento para avaliação da assistência às pessoas com comportamento suicida por profissionais da atenção primária à saúde.

**MÉTODOS:**

Estudo metodológico iniciado com revisão de literatura e construção de um instrumento que, em sua primeira versão, continha 34 itens divididos em quatro domínios: “identificação profissional”, “percepção profissional”, “conhecimento/habilidade profissional” e “organização da rede de atenção”. O conteúdo foi validado pela técnica Delphi. A análise semântica foi realizada por profissionais de nível superior de maior e menor habilidades da atenção primária à saúde. Para a análise da consistência interna, foi calculado o coeficiente alfa de Cronbach. O estudo foi realizado entre janeiro e dezembro de 2017.

**RESULTADOS:**

Após quatro rodadas Delphi, o instrumento foi validado com 50 itens, divididos em cinco domínios: “identificação profissional”, “percepção profissional”, “experiência profissional”, “conhecimento/habilidade profissional” e “organização da rede de atenção”. Os últimos quatro domínios foram inseridos em escala Likert de cinco pontos. A análise semântica apresentou 93,6% de avaliações “bom” e “muito bom”. O alfa de Cronbach geral do instrumento foi de 0,90.

**CONCLUSÕES:**

O instrumento final mostrou-se capaz de avaliar o que se propõe. Pode auxiliar em pesquisas epidemiológicas e no planejamento de ações. A avaliação da abordagem profissional ao comportamento suicida é crucial para a organização dos serviços na atenção primária à saúde e para a integração dos diferentes pontos assistenciais de atenção.

## INTRODUCTION

Suicidal behavior is typified into four different categories: suicidal ideation, suicide planning, suicide attempt, and suicide. Suicide stems from an association between biological, genetic, psychological, social, environmental, and situational factors. No single factor can provide a complete causal explanation for suicide ^1^ .

This behavior is often related to an individual’s emotional impossibility of recognizing alternatives for the resolution of conflicts and sufferings. There are several factors associated with the risk of suicide, including disabling physical illnesses, mental illnesses, abuse of alcohol and other drugs, as well as socioeconomic and family issues ^1 , 2^ .

Although it is considered preventable, worldwide more than 800,000 people commit suicide every year. It is estimated that for each effective act of suicide, there are more than 20 attempts ^1^ . Brazil is the eighth country in absolute number of suicides, with almost 12,000 annual deaths (approximately 32 per day) ^1^ . From 2004 to 2014, there was a higher concentration of suicides among individuals aged 20 to 49 years, as well as a 47% increase in the number of suicides among older people in the 60–69 age group ^3^ .

In a survey conducted in England, 91% of the sample had consulted a doctor at their primary health care provider (PHC) at least once in the year prior to the suicide act; almost 50% had their last consultation with this professional in the month prior to suicide, and one sixth in the week prior ^4^ .

The current suicide and attempted suicide rates point to a serious public health problem, which requires preventive action. In view of this scenario, Brazil has instituted National Guidelines for the Prevention of Suicide. The document recognizes the importance of preventative interventions, highlighting measures such as the organization of care networks for people at risk of suicide, as well as the development of data collection and analysis methods that enable the sharing of information and knowledge. It also suggests the promotion of continuing education for health professionals, including in the PHC ^5^ . The training of PHC teams to identify, approach, manage and refer people with suicidal behavior is an important means of prevention, since these professionals’ proximity and bonds with the population are the main resources of the primary health care system ^6^ .

An understanding of these professionals’ practice is fundamental, and having instruments that provide valid and reliable measures to identify their competencies is a relevant factor for building guidelines and organizing the care network ^7 , 8^ . Identifying the knowledge and practice of professionals working in the PHC system allows for the design and development of more effective training, subsidizing the planning of health interventions.

In the literature review, we found no studies on instruments to evaluate the care for suicidal people provided by these professionals. In view of the above, the objective of the study was to construct and validate an instrument for assessing the care provided by college-educated PHC professionals to people with suicidal behavior.

## METHODS

This was a methodological study developed in four phases, describing the instrument’s construction process, the validation of its contents by specialists, the semantic analysis of its questions, and the evaluation of its internal consistency. The research was conducted between January and December 2017.

In the construction phase, the scientific literature was reviewed. Descriptors used in the search were: health personnel, suicide, suicide attempted, scales, primary health care, surveys and questionnaires, and delivery of health care, in English and Portuguese languages. The Web of Science, PubMed, and Medline databases were consulted. Inclusion criteria considered complete publications in national and international journals, starting from 1990. After reading of the abstracts, studies that did not fit this criteria were excluded. All selected articles were read in two stages: I. search for relevant suicide prevention aspects, and confirmation of inclusion in the study; and II. selection of information on the subject for inclusion in the instrument. A total of 159 articles was found; 151 were excluded. Thus, the elaboration of the instrument was based on the consultation of eight articles, as well as on the recommendations and protocols of the World Health Organization ^1 , 6^ , the Ministry of Health ^a , b^ , and the Brazilian Association of Psychiatry ^c^ . The selected articles referred to instruments for the assessment of professional attitudes toward suicidal behavior, but none focused on the PHC system or on the assessment of professional care itself.

Based on the literature review, the first version of the instrument was elaborated, consisting of 34 items, among which eight concerned professional characterization and 26 were aimed at the investigation of professional sensibility and knowledge, as well as the organization of the care network. Items were divided into four domains: “professional characterization,” “professional sensibility,” “professional knowledge/abilities,” and “organization of the care network.” Items regarding the knowledge and practice of professionals were answered according to a five-point Likert scale, with five representing maximum agreement, and 1 representing minimum agreement.

In the second phase, the content of the instrument was validated by specialist judges. Participation in the research as a judge entailed the following requirements: I. to work in the area of mental health or in the PHC network, having been appointed by the Ministry of Health, State Health Department of Mato Grosso do Sul, Municipal Health Secretariat of Campo Grande, Brazilian Association of Psychiatry, or Oswaldo Cruz Foundation (Fiocruz); or, II. to have scientific publications in the area of mental health or PHC. Specialists who had been working in their respective functions for less than a year, or who did not respond to the invitation to participate within the established deadline, were excluded. The Delphi method was used, with specialists being consulted about the instrument’s proposals. Consensus required at least two Delphi rounds ^9^ .

The survey began with seven judges. Six answered it ^10^ , remaining for four Delphi rounds. The instrument was sent via e-mail or traditional mail for item-by-item evaluation. Each judge gave their opinion on whether items should remain as they were, inserted, changed, or removed. Judges also provided their general evaluation of the instrument. Answers were regarded as stable when there was an agreement of at least 70% between judges ^11^ . The researchers proposed a duration of up to 21 days for each round, including 14 days for analysis and feedback by the group of judges, and a maximum of seven days for a response by the researcher, with a new version of the instrument attached, at which point the new round could begin. Answers and analysis were provided by the judges in an Excel ^®^ spreadsheet. Whether or not consensus was reached, the outcome of the previous round was reported to the group during the evaluation of the new iteration (which included the proposed changes).

This process led to a final version containing 50 items, with 14 belonging to the “professional characterization” domain, and 36 belonging to the four other domains: “professional sensibility,” “professional experience,” “professional knowledge/abilities,” and “organization of the care network.”

It is worth noting that the domain “professional characterization” differs from others, since it could not be answered according to a Likert scale. Analyzing the internal consistency of its items was not possible. However, according to the body of judges, this domain is extremely important for the instrument’s objectives. This is because it is not limited to the characterization of sociodemographic profile, but evidences data referring to professionals’ training and practices in dealing with risk factors for suicidal behavior. The researchers agreed with the judges, so the domain was kept in the instrument’s final version.

Content validation was followed by the research’s third phase: semantic analysis. This step examined whether the target population – including strata of greater or lesser ability ^10^ – was able to comprehend all items. College-educated professionals who worked in the Family Health Strategy (ESF), Basic Health Units (UBS) or *Consultório na Rua* (Doctor’s Office in the Street) were considered as belonging to the stratum of lesser ability. Professionals of the Family Health Support Centers (NASF) were considered as belonging to the target population’s stratum of greater ability.

The inclusion criteria were: I. to work in the municipal health network performing a function within the professional’s area of graduation; and II. to have an electronic address for receiving the link to access the evaluation. Professionals who had been in PHC for less than six months or who did not fill the questionnaires until the end of the process were excluded from the survey.

The open source software LimeSurvey was used. Participants accessed a web page where they were able to fill out the informed consent form (TCLE), the judge-validated instrument, and the semantic analysis questionnaire. The page was sent individually, via e-mail, to 283 professionals, who had ten days to participate. Fifty-three professionals participated, among whom three were excluded because they had been working in the PHC system for less than six months. Fifty (50) remained.

Cronbach’s alpha was calculated from answers given by the professionals, according to a Likert scale, to items in four domains. The items were related to assistance provided to people with suicidal behavior, the study’s object. This test checks responses’ pattern consistency as well as the instrument’s reliability, and its result should be equal to or greater than 0.70 ^12^ . Descriptive statistics were performed using SPSS software version 24.0. Results are presented descriptively and in tables.

The research was approved by the Research Ethics Committee of the Federal University of Mato Grosso do Sul, through Opinion 1,843,583, (November 29, 2016). Expert judges and all PHC professionals who participated in the research signed the informed consent term.

## RESULTS

Six judges participated in the application of the Delphi technique. Their characterization is shown in Table 1 . In the first round, a consensus of 70% was obtained for 21 (61.7%) of the instrument’s 34 items. The domain “professional characterization” reached 75% consensus; “professional sensibility,” 87.5%; “professional knowledge/abilities,” 50%; “organization of the care network,” 37.5%.

**Table 1:** Sociodemographic and training variables of judges who participated in the Delphi rounds. Campo Grande, state of Mato Grosso do Sul, Brazil, 2017.

Variable	% (n), or mean (SEM)
Sex	
Female	66.7 (4)
Male	33.3 (2)
Age (34 to 69 years)	50.50 (5.88)*
Occupation	
Doctor	66.7 (4)
Psychologist	33.3 (2)
Graduation completion time	
Up to 20 years	33.3 (2)
More than 20 years	66.7 (4)
Graduate education	
Specialization	83.3 (5)
Master’s Degree	66.7 (4)
Doctorate	16.7 (1)
Field of practice	
Health care	66.7 (4)
Teaching-research	66.7 (4)
Administration	50.0 (3)

SEM: standard error of the mean

* Values for mean and SEM.

At the end of the first round, the writing of 14 questions had to be modified: two in the “professional characterization” domain, one in “professional sensibility,” eight in “professional knowledge/abilities,” and three in “organization of the care network.” The domain “professional experience” was added, and nine new questions were inserted, two in “professional sensibility” and seven in “professional experience.” The second version of the instrument had 43 items and five domains.

In the second round, a consensus of 70%, in 41 (95.3%) of the instrument’s 43 items, was obtained. The domains “professional characterization” and “professional sensibility” presented 100% consensus; “professional experience” obtained 85.7%; “professional knowledge/abilities,” 90%; and “organization of the care network,” 100%.

Seven questions had their wording changed, one in the “professional characterization” domain, three in “professional sensibility,” two in “professional experience,” and one in “professional knowledge.” Seven questions were inserted, six in the “professional characterization” domain and one in the “professional knowledge/abilities” domain. This third version of the instrument was comprised of 50 items. The third round reached a consensus of 70% in 49 (98%) of the 50 items. Only one item, belonging to the “professional characterization” domain, had a lower level of agreement (67%).

In the fourth round, we asked for an evaluation of the only question that had not obtained a consensus, with the inclusion of information on the item’s subject. The result was an agreement of 83%. The instrument was entitled Instrument for the Evaluation of Professional Assistance to People with Suicidal Behavior (IAAP-PCS). Its 50 items are divided into five domains: “professional characterization,” “professional sensibility,” “professional experience,” “professional knowledge/abilities” and “organization of the care network.”

A five-point Likert-type scale was used for answering each item, except in the “professional characterization” domain. Data related to the maintenance, addition and modification of items are presented in the Box .

**Box:** Changes made in the instrument (assessment of care provided by college-educated professionals of primary health care to people with suicidal behavior). Campo Grande, state of Mato Grosso do Sul, Brasil, 2017.

First version	Final version	Action
*Domain I: professional characterization*	*Domain I: professional characterization*	No change
1) Profession	1) Profession	No change
2) Sex: ( ) Female ( ) Male	2) Sex: ( ) Female ( ) Male	No change
3) Age	3) Age	No change
4) Specialization: ( ) Yes ( ) No. If you answered yes, which one?	**4) Postgraduate degree: ( ) Yes ( ) No. If you answered yes, which one?**	**Changed**
5) Working time in the current health unit (months)	5) Working time in the current health unit (months)	No change
6) Are you an on-call attendant in urgent care/emergency services (emergency care units – ECU, hospital emergency room, or similar)? ( ) Yes ( ) No	6) Are you an on-call attendant in urgent care/emergency services (emergency care units – ECU, hospital emergency room, or similar)? **( ) Yes ( ) No. If you answered “yes,” please specify:**	**Changed**
7) In your professional education, did any of your courses discuss the subject of suicide? ( ) Never ( ) A few times ( ) Sometimes ( ) I do not remember ( ) Many times ( ) Always	7) During your professional education, how many courses dealt with the subject of suicide? **( ) None ( ) 1 to 2 ( ) 3 to 4 ( ) 5 or more ( ) I do not remember**	**Changed**
8) Have you ever participated in any workshop, seminar, lecture or congress on the subject of “suicide”? ( ) Yes ( ) No. If you answered “yes,” in which year(s)?	8) Have you ever participated in any workshop, seminar, lecture or congress on the subject of “suicide”? ( ) Yes ( ) No. If you answered “yes,” in which year(s)?	No change
-	**9) Have you received training on mental health issues in the last 12 months? ( ) Yes ( ) No**	**Item added**
-	**10) How often do you treat people with disabling physical illnesses? ( ) Never ( ) Few times ( ) Sometimes ( ) Many times ( ) Always ( ) I do not remember**	**Item added**
-	**11) How often do you treat people with chronic pain? ( ) Never ( ) Few times ( ) Sometimes ( ) Many times ( ) Always ( ) I do not remember**	**Item added**
-	**12) How often do you treat people with mental disorders? ( ) Never ( ) Few times ( ) Sometimes ( ) Many times ( ) Always ( ) I do not remember**	**Item added**
-	**13) How often do you assist people aged 60 years or over? ( ) Never ( ) Few times ( ) Sometimes ( ) Many times ( ) Always ( ) I do not remember**	**Item added**
-	**14) How often do you assist people aged 15 to 30 years? ( ) Never ( ) Few times ( ) Sometimes ( ) Many times ( ) Always ( ) I do not remember**	**Item added**
*Domain II: professional sensibility*	*Domain II: professional sensibility*	No change
1) I believe that suicide is a public health problem	1) I believe that suicide is a public health problem	No change
2) I am able to identify a person with suicidal behavior	**2) I am able to identify people with suicidal behavior**	**Changed**
3) I believe suicide can be prevented	3) I believe suicide can be prevented	No change
4) I understand that talking about it is one of the ways to avoid suicide	**4) I understand that talking about it is one of the ways to prevent suicide**	**Changed**
5) I, a primary care practitioner, can help with suicide prevention	5) I, a primary care practitioner, can help with suicide prevention	No change
6) I believe that it is the attribution of the health service to monitor people who have attempted suicide	**6) I believe that it is the attribution of the health service to monitor people with suicidal behavior**	**Changed**
7) I believe that it is the attribution of primary health care to monitor people who have attempted suicide	**7) I believe that it is the attribution of primary health care to monitor people at risk of suicide**	**Changed**
8) I believe that it is the attribution of specialized health care to monitor people who have attempted suicide	**8) I believe it is the attribution of specialized health care to monitor people with suicidal behavior**	**Changed**
-	**9) I believe that the health service should monitor and provide guidance to the family of the person at risk of suicide**	**Item added**
-	**10) Suicide can be understood as a form of violence**	**Item added**
-	*Domain III: professional experience*	**Item added**
-	**11) I have provided assistance to people who attempted suicide**	**Item added**
-	**12) I tried to obtain information on whether there were previous attempts, and how many**	**Item added**
-	**13) I have followed up with people who attempted suicide (by means of home visits, phone calls and/or appointments at the health unit)**	**Item added**
-	**14) I have followed up with family members of people who attempted suicide (by means of home visits, phone calls and/or appointments at the health unit)**	**Item added**
-	**15) I have previously referred people with suicidal behavior to specialized care**	**Item added**
-	**16) I have previously referred relatives of people who attempted suicide to specialized care**	**Item added**
-	**17) I have previously registered suicide attempts via an interpersonal/self-harm reporting form**	**Item added**
**Domain III: professional knowledge/abilities**	**Domain IV: professional knowledge/abilities**	**Changed**
9) I feel qualified to address suicide prevention	**18) I am qualified to practice suicide prevention**	**Changed**
10) I feel qualified to assist someone who has attempted suicide	**19) I am qualified for providing immediate care to someone who has attempted suicide**	**Changed**
11) I feel qualified for approaching and following up with people who have attempted suicide	**20) I am qualified for approaching and following up with people who have attempted suicide**	**Changed**
-	**21) I record information regarding a suicide attempt, even if it is not the main complaint in the case**	**Item added**
12) I understand that people with mental disorders are more likely to attempt suicide	**22) People with mental disorders are more likely to attempt suicide**	**Changed**
13) I understand that a previous suicide attempt is a risk factor for a new attempt	**23) A previous suicide attempt is a risk factor for a new attempt**	**Changed**
14) I perform home visits, as they can help in the prevention of suicide	**24) Home visits can help in the prevention of suicide**	**Changed**
15) I maintain regular communications (phone calls, mobile text messages – SMS or WhatsApp), containing suicide prevention guidelines regarding people who have attempted suicide	**25) Regular communications (phone calls, mobile text messages – SMS or WhatsApp) on suicide prevention guidelines help health professionals work with people who have attempted suicide**	**Changed**
16) I provide guidance to relatives and friends of people who have attempted suicide, regarding precautionary measures and prevention of further attempts (e.g. restricting access to lethal means, maintaining care and dialogue, evaluating, monitoring and maintaining treatment)	**26) Guidance to family members and friends of people who have attempted suicide, regarding precautionary measures and prevention of further attempts (e.g. restricting access to lethal means, maintaining care and dialogue, evaluating, monitoring and maintaining treatment) helps prevent suicide**	**Changed**
17) I follow up with people who have attempted suicide (by means of home visits, phone calls and/or appointments at the health unit), because I believe I can help them	**27) Follow-up of people who have attempted suicide (by means of home visits and/or appointments at the health facility) helps prevent suicide**	**Changed**
18) I understand that the health service should work together with other sectors, such as social assistance, education, churches, NGOs and the media, for preventing suicide	**28) The health service should work together with other sectors, such as social assistance, education, churches, NGOs and the media, for the prevention of suicide**	**Changed**
**Domain IV: organization of the care network**	**Domain V: organization of the care network**	**Changed**
19) At the health unit where I work, there are professionals prepared to assist people with suicidal behavior	29) At the health unit where I work, there are professionals prepared to assist people with suicidal behavior	No change
20) At the health unit where I work, there is physical structure to assist people with suicidal behavior	**30) At the health unit where I work, there is a welcoming environment and a private room to assist people with suicidal behavior**	**Changed**
21) At the health unit where I work, there are drug resources to assist people with suicidal behavior	31) At the health unit where I work, there are drug resources to assist people with suicidal behavior	No change
22) At the health unit where I work, our team receives specialist orientation by teams from reference services	**32) At the health care unit where I work, our team receives specialist orientation by teams from reference services (e.g. CAPS and NASF)**	**Changed**
23) I am able to refer someone who has attempted suicide to specialized services within 24 to 48 hours	33) I am able to refer someone who has attempted suicide to specialized services within 24 to 48 hours	No change
24) I am informed when a person who has attempted suicide, and who resides in my area of assignment, is discharged from the specialized service	34) I am informed when a person who has attempted suicide, and who resides in my area of assignment, is discharged from the specialized service	No change
25) I am informed by the specialized service about the treatment, with drugs or otherwise, prescribed to people who have attempted suicide while residing in my area of assignment	35) I am informed by the specialized service about the treatment, with drugs or otherwise, prescribed to people who have attempted suicide while residing in my area of assignment	No change
26) The municipal public health service collaborates with other sectors, such as social assistance, education, churches, NGOs and the media, for preventing suicide	**36) The municipal public health service in which I work collaborates with other sectors, such as social assistance, education, churches, NGOs and the media, for the prevention of suicide**	**Changed**

SMS: short message service; NGOs: non-governmental organizations; CAPS: Center for Psychosocial Care; NASF: Family Health Support Center

Excerpts in bold refer to changes.

The characterization of the professionals who participated in the semantic analysis is shown in Table 2 . It indicates that the instrument fulfilled its goals, receiving 93.6% “good” and “very good” evaluations.

**Table 2:** Sociodemographic and educational variables of professionals who participated in semantic analysis. Campo Grande, state of Mato Grosso do Sul, Brazil, 2017.

Variable	% (n), or mean (SEM)
Sex	
Female	86.0 (43)
Male	14.0 (7)
Age (27 to 60 years)	39.92 (1.46)*
Sanitary district	
North	56.0 (28)
South	26.0 (13)
West	16.0 (8)
East	2.0 (1)
Workplace	
ESF/UBS/ *Consultório na Rua*	72.0 (36)
NASF	28.0 (14)
Occupation	
Nurse	40.0 (20)
Social worker	16.0 (8)
Physical educator	8.0 (4)
Pharmacist	6.0 (3)
Doctor	6.0 (3)
Psychologist	6.0 (3)
Physical therapist	4.0 (2)
Speech therapist	4.0 (2)
Nutritionist	4.0 (2)
Dentist	4.0 (2)
Occupational therapist	2.0 (1)
Service time in the current health unit (2 to 204 months)	43.10 (6.36)*
Service time in primary health care (9 to 324 months)	78.64 (10.33)*
On-call attendee in urgent/emergency services	
Yes	58.0 (29)
No	42.0 (21)
During your professional education, how many courses dealt with the subject of suicide?	
None	26.0 (13)
1 to 2	54.0 (27)
5 or more	6.0 (3)
Did not remember	14.0 (7)
Have you ever participated in any workshop, seminar, lecture or congress on the subject of “suicide”?	
Yes	60.0 (30)
No	40.0 (20)
Have you received training on mental health issues in the last 12 months?	
Yes	30.0 (15)
No	70.0 (35)

ESF: Family Health Strategy; UBS: Basic Health Unit; NASF: Family Health Support Center; SEM: standard error of the mean

* Values for mean and SEM.

The instrument’s scale of responses was considered appropriate by 82% of the professionals, and 96% of the participants believed it was able to fulfill its evaluation purposes. According to 98%, the items are properly grouped and objectively formulated; all considered the instrument coherent. According to 88% of the professionals, items were easy to read and comprehend, as shown in the Figure .

**Figure:**
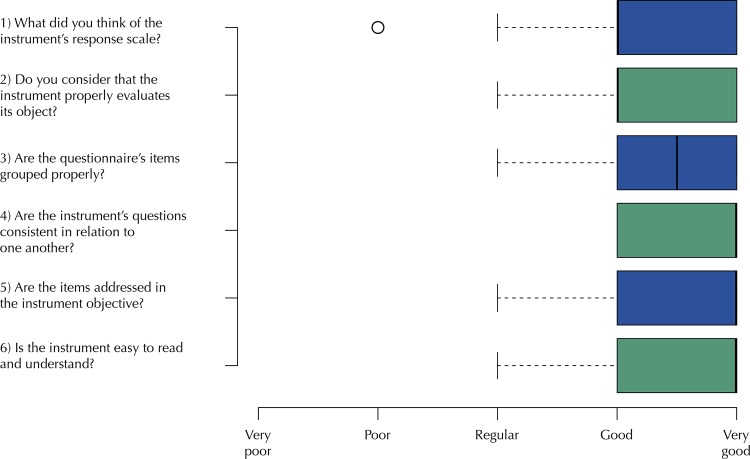
Results of semantic analysis performed by PHC professionals. Campo Grande, state of Mato Grosso do Sul, Brazil, 2017.

The general Cronbach’s alpha of the questionnaire was 0.90 (excellent internal consistency), with 0.50 (poor internal consistency) in the “professional sensibility” domain, 0.90 (excellent internal consistency) in “professional experience,” 0.82 (good internal consistency) in “professional knowledge/abilities,” and 0.73 (acceptable internal consistency) in “organization of the care network.” Table 3 presents the results of the internal consistency analysis of each domain’s items.

**Table 3:** Median and internal consistency test (for each item of the instrument). Campo Grande, state of Mato Grosso do Sul, Brazil, 2017.

Domain	Questions	Median (min. to max.)	Cronbach’s α (domain, excluding the question)	Cronbach’s α (general, excluding the question)
Professional sensibility (Cronbach’s α: 0.50)	I believe that suicide is a public health problem	5 (4 to 5)	0.48	0.90
	I am able to identify people with suicidal behavior	4 (1 to 5)	0.53	0.89
	I believe suicide can be prevented	5 (2 to 5)	0.45	0.90
	I understand that talking about it is one of the ways to prevent suicide	5 (4 to 5)	0.52	0.90
	I, a primary care practitioner, can help with suicide prevention	5 (3 to 5)	0.44	0.90
	I believe that it is the attribution of the health service to monitor people with suicidal behavior	5 (2 to 5)	0.44	0.90
	I believe that it is the attribution of primary health care to monitor people at risk of suicide	5 (2 to 5)	0.36	0.90
	I believe it is the attribution of specialized health care to monitor people with suicidal behavior	5 (2 to 5)	0.55	0.90
	I believe that the health service should monitor and guide the family of the person at risk of suicide	5 (2 to 5)	0.44	0.90
Professional experience (Cronbach’s α: 0.90)	I have provided assistance to people who attempted suicide	5 (1 to 5)	0.90	0.90
	I tried to obtain information on whether there were previous attempts, and how many	5 (1 to 5)	0.88	0.89
	I have followed up with people who attempted suicide (by means of home visits, phone calls and/or appointments at the health unit)	5 (1 to 5)	0.88	0.89
	I have followed up with family members of people who attempted suicide (by means of home visits, phone calls and/or appointments at the health unit)	5 (1 to 5)	0.88	0.89
	I have previously referred people with suicidal behavior to specialized care	5 (1 to 5)	0.88	0.89
	I have previously referred relatives of people who attempted suicide to specialized care	4 (1 to 5)	0.87	0.89
	I have previously registered suicide attempts via an interpersonal/self-harm reporting form	3 (1 to 5)	0.92	0.89
Professional knowledge/abilities (Cronbach’s α: 0.82)	I am qualified to practice suicide prevention	4 (1 to 5)	0.77	0.89
	I am qualified for providing immediate care to someone who has attempted suicide	4 (1 to 5)	0.76	0.89
	I am qualified for approaching and following up with people who have attempted suicide	4 (1 to 5)	0.76	0.89
	I record information regarding a suicide attempt, even if it is not the main complaint in the case	4 (1 to 5)	0.80	0.89
	People with mental disorders are more likely to attempt suicide	5 (1 to 5)	0.83	0.90
	A previous suicide attempt is risk factor for a new attempt	5 (3 to 5)	0.82	0.90
	Home visits can help in the prevention of suicide	5 (3 to 5)	0.82	0.90
	Regular communications (phone calls, mobile text messages – SMS or WhatsApp) on suicide prevention guidelines help health professionals work with people who have attempted suicide	4 (1 to 5)	0.82	0.90
	Guidance to family members and friends of people who have attempted suicide, regarding precautionary measures and prevention of further attempts (e.g. restricting access to lethal means, maintaining care and dialogue, evaluating, monitoring and maintaining treatment) helps prevent suicide	5 (3 to 5)	0.82	0.90
	Follow-up of people who have attempted suicide (by means of home visits and/or appointments at the health facility) helps prevent suicide	5 (4 to 5)	0.82	0.90
	The health service should work together with other sectors, such as social assistance, education, churches, NGOs and the media, for the prevention of suicide	5 (4 to 5)	0.82	0.90
Organization of the care network (Cronbach’s α: 0.73)	At the health unit where I work, there are professionals prepared to assist people with suicidal behavior	4 (1 to 5)	0.69	0.90
	At the health unit where I work, there is a welcoming environment and a private room to assist people with suicidal behavior	4 (1 to 5)	0.69	0.90
	At the health unit where I work, there are drug resources to assist people with suicidal behavior	3 (1 to 5)	0.76	0.90
	At the health care unit where I work, our team receives specialist orientation by teams from reference services (e.g. CAPS and NASF)	4 (1 to 5)	0.71	0.90
	I am able to refer someone who has attempted suicide to specialized services within 24 to 48 hours	4 (1 to 5)	0.71	0.89
	I am informed when a person who has attempted suicide, and who resides in my area of assignment, is discharged from the specialized service	3 (1 to 5)	0.70	0.90
	I am informed by the specialized service about the treatment, with drugs or otherwise, prescribed to people who have attempted suicide while residing in my area of assignment	2 (1 to 5)	0.67	0.90
	The municipal public health service in which I work collaborates with other sectors, such as social assistance, education, churches, NGOs and the media, for the prevention of suicide	4 (1 to 5)	0.69	0.90
Instrument’s general Cronbach’s α				0.90

CAPS: Center for Psychosocial Care; NASF: Family Health Support Center; NGOs: non-governmental organizations

The instrument innovates in establishing the evaluation of professional health care practice in the face of suicidal behavior, with easily understood items and a Likert-type scale of responses. The “professional characterization” domain covers the professional’s training and qualification, time of service, and how frequently the professional encounters risk factors for suicide. The “professional sensibility” domain provides knowledge on how the professional perceives and understands the importance of the health service in the context of assisting people with suicidal behavior, constituting an important tool in a future process of sensitization. “Professional experience,” a fundamental component, assesses how seasoned a professional is in certain activities, as higher levels of experience can benefit the provided assistance. Theoretical-practical knowledge makes it possible to provide assistance based on efficient health policies and reliable studies; this information is evaluated in the “professional knowledge/abilities” domain. Finally, for the provision of care, in addition to the professional’s activity, a physical and medical structure is necessary, together with established work processes aimed at the formation of organized and efficient care networks. The “organization of care networks” domain aims to verify the local network’s readiness to act in the prevention of suicide.

## DISCUSSION

The IAAP-PCS instrument has the potential to enhance the integration of mental health-related care into the PHC system, since it allows assessing and identifying problematic aspects of care, as well as consolidated practices. It may also be useful for pointing out possible vulnerabilities and gaps in the country’s mental health policy, in regards to the preparation of PHC professionals.

As in many countries, in Brazil this integration is occurring gradually, with a view to strengthening the primary care network and overcoming the country’s history of unwarranted institutionalization practices targeting the mentally ill ^13^ . However, this process is not always accompanied by strategies for the evaluation of the provided services. This type of evaluation is essential for obtaining knowledge on past and current practices in the field of mental health, especially at this level of care ^14^ .

This instrument, designed to be applied to professionals working in PHC, can be an important tool in the development of best-practices for dealing with suicidal behavior, contributing to the field of epidemiology. The potential of the instrument to be used at both the individual and collective levels is in line with the stance advocated by several scholars, who point out that one of the main challenges is the inclusion of mental health care in the context of PHC, as part of a set of individual and collective, community-based actions ^15^ .

The lack of instruments to address suicidal behavior in PHC has led to the elaboration of the IAAP-PCS ^18^ . The instrument’s domains are in accordance with World Health Organization policy and the protocols adopted by the Brazilian Ministry of Health. In the construction of the instrument, aspects related to suicide prevention strategies were taken into consideration – such as surveillance measures, identification of risk factors and vulnerable groups – as well as the importance of health professionals, especially those in PHC, given their direct contact with local health demands, which facilitates the identification of people with suicidal behavior. These strategies are also aligned with the proposals of scholars who research the thematic of care for people with suicidal behavior. Those scholars emphasize that mental health actions in the PHC should be directed to the promotion of health, prevention of aggravations and general treatment, considering the demands of the territory and emphasizing community participation in the processes of planning, operationalization and control ^15^ .

College-educated professionals were chosen to respond to the instrument due to their ability to identify risk factors, and also due to their involvement in follow-up ^19^ . It is important for practitioners to have the competence to understand their role in the process, and that they are prepared to act accordingly. Research indicates that professionals do not consider themselves prepared for this type of care and act primarily by referring patients to specialized services. In fact, most of the mental health actions identified consisted of team meetings, professional training and articulation between PHC and specialized services ^14^ . The organization of the instrument consisted of domains that allow identifying potential areas of improvement in the perception and approach of the problem, making it possible to assess the preparation of PHC professionals in dealing with suicidal behavior, and to discuss their role.

The Delphi method is the most appropriate for instrument validation, since it seeks the consensus of professionals with great expertise in the field. Studies indicate that a 70% or higher level of consensus, the cut-off point used in this research, is enough to qualify the instrument ^20^ .

In the present study, after the Delphi rounds, semantic analysis was done with professionals who were part of the target population. In similar studies, this type of analysis has proven to be an important addition to instruments’ validation process. One of these studies performed semantic analysis in order to verify if the items were intelligible to a meta-population of 15 technicians and nursing auxiliaries ^21^ . In another study, a scale was used to measure recovery in eight patients who had undergone intensive treatment. The patients themselves participated in the instrument’s semantic analysis ^25^ . In the construction and validation of an instrument focused on good practices in the care of normal births, the semantic analysis was performed by eight health professionals, who again were part of the instrument’s target population ^26^ . In our study, semantic analysis did not lead to changes in the instrument, since the respondent target population had a positive evaluation ^25 , 26^ .

As for internal consistency, the 0.90 value observed here is similar to results of other studies, which ranged from 0.87 to 0.91 ^27 , 28^ . In several studies, a consistency of 0.60 to 0.80 was also considered satisfactory ^20 , 29^ .

One limitation of the study concerns the semantic analysis step: it would be preferable for this stage to incorporate professionals of the entire health care network, so as to obtain an understanding on the system’s mechanisms of communication, and on the interventions performed across the assistance network. We emphasize that, although the semantic analysis methodology adopted in this study recommends items to be verified from the perspective of strata of greater and lesser skill in the instrument’s target population, no such analyzes were performed, since this was not the object of study.

The elaboration of the domains and its items started from an exhaustive search in the literature, in order to find scientific evidence about usual practices and what is necessary to make PHC assistance more qualified and adequate in the prevention of suicides. The employed methodological procedures (literature review, choice of the Delphi method to validate the instrument based on expert knowledge, semantic analysis for obtaining a broad intelligibility of the instrument, and analysis of internal consistency by Cronbach’s alpha) were adequate for evaluating an instrument to assess the assistance provided to the people with suicidal behavior, qualifying the care network.

The IAAP-PCS can aid in epidemiological research and in the planning of actions to foster the practice of assessing the care provided to individuals with suicidal behavior, establishing agile and interconnected forms of care.

## Footnotes

a Ministério da Saúde (BR), Secretaria de Atenção à Saúde, Departamento de Atenção Básica. Saúde mental. Brasília (DF); 2013. (Cadernos de Atenção Básica; n. 34). Available from: http://bvsms.saude.gov.br/bvs/publicacoes/cadernos_atencao_basica_34_saude_mental.pdf

b Ministério da Saúde (BR). Prevenção do suicídio: manual dirigido aos profissionais da saúde da atenção básica. Brasília (DF); 2009. Available from: http://portal.saude.gov.br/portal/arquivos/pdf/manu_prevencao240111.pdf

c Associação Brasileira de Psiquiatria. Suicídio: informando para prevenir. Brasília, DF: CFM/ABP; 2014. Available from: https://www.cvv.org.br/wp-content/uploads/2017/05/suicidio_informado_para_prevenir_abp_2014.pdf
